# Children's Green Infrastructure: Children and Their Rights to Nature and the City

**DOI:** 10.3389/fsoc.2022.804535

**Published:** 2022-04-04

**Authors:** Diogo Guedes Vidal, Eunice Castro Seixas

**Affiliations:** ^1^Centre for Functional Ecology, TERRA Associate Laboratory, Department of Life Sciences, University of Coimbra, Calçada Martim de Freitas, Coimbra, Portugal; ^2^Faculty of Science and Technology, University Fernando Pessoa, Porto, Portugal; ^3^Research Centre in Economic and Organizational Sociology (SOCIUS), Research in Social Sciences and Management (CSG), Lisbon School of Economics and Management (ISEG), Universidade de Lisboa, Lisbon, Portugal

**Keywords:** children's rights, salutogenesis, inclusive urban planning, green infrastructure, Urban green space

## Abstract

The development of green spaces in cities has corresponded to a need to deal with a series of socio-environmental and health problems felt in urban spaces. However, these are often fragmented or somewhat disconnected interventions that leave out vulnerable and subaltern groups like children, being also commonly based on strictly formatted designs, with more urban furniture than natural elements. In view of the need to make urban spaces healthier, safer, more resilient, and at the same time more child-friendly, in this Conceptual Analysis paper we build from the literature on Urban Green Spaces, Child-Friendly Cities and environments, and Children's Infrastructure to propose the concept of Children Green Infrastructure (CGI), and discuss its application to urban planning, foregrounding the need for fairer, more inclusive and participatory approaches. GGI derives from the Children Infrastructure concept but it puts at the center of the debate the idea of connecting children to nature where they live, learn and play. CGI is based on the assumption that nature should be transversal in urban planning processes, and that it must be perfectly integrated within urban infrastructures, ensuring access to all. Understanding children's needs and integrating their voices in urban planning and design processes are necessary conditions to moving forward to a fairer, more inclusive and truly collective urban project.

## Introduction

Significant evidence has pointed out the multiple benefits of Urban Green Spaces (UGS). Much of the debate has been developed on the ecosystem services framework provided by UGS (Haines-Young and Potschin, 2018), related to provision (Kazemi et al., 2018), regulation and maintenance (Mathey et al., 2011; Graça et al., 2018) and cultural services (Jennings et al., 2016; Liotta et al., 2020). Nature exposure also contributes to making us more “immune” to urban stressors, improving mental health outcomes (Vidal et al., 2020b; Lencastre and Farinha Marques, 2021). Beyond these multiple benefits, UGS have been proven to be an avenue to health promotion, namely in deprived communities, also by enhancing social cohesion and a sense of belonging (Jennings and Bamkole, 2019). It is widely acknowledged that urban spaces face socio-environmental inequities, affecting the most vulnerable groups (Hoffimann et al., 2017; Vidal et al., 2021a). Children, as a social category of generational type, are also exposed to a variety of social, economic and environmental risks, limiting their opportunities for agency and development (Mansfield et al., 2021). Although on average, urban children enjoy better access to essential services as well as to cultural, educational and other opportunities for development, this “urban advantage” masks enormous disparities and inequities among urban residents (UNICEF, 2018).

Children's access to quality UGS and their use of the same, is also subjected to these inequities, with income disparities, social class and racial and ethnic belonging all having an important role in this regard (Abercrombie et al., 2008; Boone et al., 2009; Johnson-Gaither, 2011; along with gentrification, commodification and other neoliberal processes with impact in urban planning (Karsten, 2007; Formoso et al., 2010; Karsten and Felder, 2015).^1^ Furthermore, the way that UGS are designed results frequently in a limited opportunity for children to fully explore these spaces due to the high presence of urban furniture, instead of natural elements (Woolley, 2008; Ferret, 2021; Vidal et al., 2021b). All these constraints threaten the need to ensure universal access to safe and inclusive green spaces in cities, especially for children, women and unprivileged social groups, as framed by the UN 2030 Agenda (United Nations, 2015). Indeed, children's studies have called attention to the disappearance of urban children from the public arena, relating this to processes of institutionalization, privatization and insularization of space (Zeiher, 2003; Leverett, 2011; Sarmento, 2018) as well as to exacerbated representations of the urban public places as dangerous and children as vulnerable (Gill, 2007; Tomás, 2007). In his book, Gill (2007) presents some social statistics that illustrate this evidence: “In 1971 eight out of ten children aged seven or 8 years went to school on their own. By 1990 this figure had dropped to less than one in ten. Again, in 1971 the average 7-year-old was making trips to their friends or the shops on their own. By 1990 that freedom was being withheld until the age of ten, meaning that in just 19 years children had ‘lost' up to 3 years of freedom of movement” (Gill, 2007, p. 12). The author furthers his argument by stating that the amount of time that parents spend looking after their children “…has quadrupled in just 25 years, from 25 minutes per day in 1975 to 99 minutes in 2000, and one of the reasons for this is a fear of letting children play unsupervised” (Gill, 2007, p. 13). On the other hand, recent literature has revealed middle-class preferences for an “urban lifestyle” that includes practices of “family outing” and of “consuming the city” and their public spaces by children and their families (Karsten, 2007; Karsten and Felder, 2015).

Given this evidence, it is undeniable that cities must be planned with and for children, and even more when it is projected that by 2030 more than 60% of the population residing in urban areas will be under 18 years of age (UNICEF, 2018). However, although the studies carried out on the topic of UGS aimed to provide up to date knowledge with the goal of improving urban environmental quality and promoting the wellbeing of the urban population, there has been a neglect of the structural deficiencies and inequalities that are constantly reproduced within the urban fabric (Jennings et al., 2021). Cities are spaces of production and reproduction of inequalities (Lefebvre, 1974), and among these, environmental inequalities are gaining expression amongst urban settlements (Laurent, 2011; Kabisch et al., 2016; Liotta et al., 2020). Moreover, cities tend to be characterized by “…adult-centric legislation, policies, rules and practices that are embedded within social structures and institutions which impact negatively on children's daily lives and result in disadvantage and oppressive social relations.” (LeFrançois, 2014, p. 517). This adultism has contributed to the invisibility of children in urban policies and undermined both the realization of children's right to the city and the quality and resilience of the urban fabric since this is also a function of the resilience of its most vulnerable social groups (Castro Seixas and Giacchetta, 2020). As argued by Cordero Arce (2012), a hegemonic children's rights discourse, promoted, in part, by the United Nations Convention on the Rights of the Child, is depicting children as passive actors where their rights are understood as an adult concession. In the same line, Skelton (2007), suggests that UNICEF's concept of children's participation, being framed in an abstract and decontextualized manner, actually contributes to rendering social inequalities and exclusion processes invisible. Thus, although the importance of the Convention on the Rights of the Child as “(…) a legal and symbolic landmark, pointing to a universality of rights for younger citizens” (Sarmento, 2020, p. 23) is indisputable, as it is its acknowledgment of children's rights to participation, there is also a need for a critical reflection on the Convention (Gillett-Swan and Thelander, 2021). As Sarmento, 2020 suggest: “it is necessary to reflect on the improvement of its effectiveness and its own content, which needs to integrate the changes that have taken place in contemporary societies and in the ways of life of children” (p. 23).

Previous studies have shown that children are not provided with the opportunities to contribute to the development of their own environment, thus becoming invisible in the landscape and forced to fit into “unfriendly environments of the adults” (Matthews, 1995; Sutton, 1996; Tsevreni, 2015; Ataol et al., 2019; Zerlina and Sulaiman, 2020). It is against this background that we argue for a shift of the paradigm and intervene in the (infra)structural dimension of the city, including the children in this process. With this goal in view, in this paper, we propose a new concept—Children Green Infrastructure (CGI), and discuss its potential application in urban planning. We suggest that this concept opens new possibilities for the realization of children's rights, namely their right to the city, their right to play and their right to nature. At the same time, CGI foregrounds the need for fairer, more inclusive and truly participatory approaches to urban planning.

## Toward Healthy, Child-Friendly, and Inclusive Cities

Healthy cities are anchored in the concept of “salutogenesis” (Antonovsky, 1979), which characterizes these as places of protection from diseases and support for the creation and maintenance of health. The recent experience of the COVID-19 pandemic has highlighted the protective role of UGS, namely those spaces that are fully accessible and provide recreational opportunities (Kleinschroth and Kowarik, 2020; Rodgers, 2020). The multiple benefits for mental health are well documented (Tendais, 2020; Mayen Huerta and Utomo, 2021; Ribeiro et al., 2021), with for example Wortzel et al. (2021) finding that youngers experienced lower COVID-19 related worries as a function of their accessibility to UGS. For children, healthy cities are places where they have the freedom to play, explore and socialize, without restrictions or constraints (Kyttä, 2004). In this regard, encouraging street play by improving the streets and spaces next to the children's homes helps promoting the integrative development of children (Thaler and Sunstein, 2008) and realizing children's right to play (Davey and Lundy, 2011). Children can find affordances for play in both formal, planned spaces and informal/unmanaged places (Jansson et al., 2016). The multifunctionality and richness of planned spaces are particularly appreciated by children, but unmanaged areas are also valued for exploration, play and creating “children's places” (Jansson et al., 2016). With Brown et al. (2019), we see “the focus on child-friendly cities as a valuable entry point for integrated healthy city commitment, policy and action, as set out at the foundation of the WHO Healthy Cities initiative” (p. 1). Thus, a child-friendly city is a city that is healthy not just for children but for all citizens.

In the wake of the U.N. General Assembly's adoption of the Convention on the Rights of the Child (CRC) in 1989, a rights-based approach to creating child-friendly environments prevailed, giving rise to several important UN initiatives such as the UNICEF Child-Friendly Cities (CFC). CFC framework builds from a holistic perspective of children's rights as comprising both their access to urban resources (rights in the city), and to meaningful participation in urban governance (rights to the city), and it has given wider visibility to the need for integrating children's rights into decision-making and city governance. A child-friendly city should promote children's rights, and provide safe spaces to play, allowing for a strong connection with nature, encouraging independent mobility and, above all, including children in the processes of reformulation and design of urban places and policies (Brown et al., 2019). UGS, especially those with public access, have the potential to promote a healthy lifestyle for children (Dadvand et al., 2019), and are important in fostering children's integrative development and wellbeing (Fjørtoft, 2004; Faber Taylor and Kuo, 2009; Ward et al., 2016; Sarmento, 2018; Neto, 2020; Fjørtoft et al., 2021; Ito, 2021). A Danish study (Engemann et al., 2018) has showed that children who lived in environments without green spaces had increased risk for psychosis, compared to those who lived in green areas. Furthermore, literature on biophilia (Wilson, 1984) has suggested that humans have a spontaneous relationship or predisposition to connect with nature, whose intensity varies according to their exposure. This hypothesis has been extended to children (Kahn, 1997; Keith et al., 2021) and supported by studies on children's development showing that throughout their childhood, children experience important milestones related to nature connectedness, which emphasize the relationship with nature in multiple ways (Neaum, 2010; Svetlova et al., 2010; Bensalah et al., 2016), namely by the interaction of children with non-human species that starts in early childhood (DeLoache et al., 2010). Nonetheless, some authors have also suggested that this nature connectedness of children can be lost or perhaps it is not innate after all and is instead learnt and depends on nature exposure (Hand et al., 2017).

In the last 10 years, other international initiatives were designed to promote the inclusion of children, their voices and perspectives in city planning: Urban95, which sought to re-imagine cities from 95 cm tall (Vincelot, 2019), or the publication “Cities alive: designing for urban childhoods” (ARUP, 2017) that put children in the spotlight to respond to the main social and environmental challenges in contemporary cities. Nevertheless, and despite its importance in giving visibility to children, their rights and capabilities, the UNICEF CFC framework has also proved to be insufficient and liable to co-option in many cases (Racelis and Aguirre, 2002, 2006). Co-option is facilitated by the fact that there is no single definition of what a child-friendly city is or ought to be, and therefore, different conceptualizations and approaches to CFC emerge within the context of neoliberal globalization. In fact, the idea of what a child-friendly city is might vary according to different cultural and socio-economic contexts. Van Vliet and Karsten's proposal (van Vliet and Karsten, 2015) is key in this respect as it reveals the different approaches to children's relationship with the city, with children being framed as consumers, users, entrepreneurs and/or producers. Moreover, although there may be common features of child-friendly environments, there are sociocultural variations in the subjective experiencing of these features and in their contribution for the resilience of children (Derr et al., 2019). The main focus of child-friendly cities initiatives also depends on the economic status of the country where it is applied, as for high-income nations the focus has been on improving the quality of spaces available for children in the city, while in low-income nations priority has been given to survival issues, including access to basic services and children's safety and security (Malone, 2016). These differences point to the importance of taking into account the context, and listening to children and their families within the process of building child-friendly cities and environments. Horelli's (2006, cit in Haikkola et al., 2007, p. 322) definition of environmental child-friendliness is relevant here as the author considers it to be “a complex multi-dimensional and multi-level concept”, which “refers to settings and environmental structures that provide support to individual children and groups who take an interest in children's issues, so that children can construct and implement their goals or projects.” Although participation of children is part of the framework, this again is no guarantee of protection against co-option and manipulation of children within the process.

When thinking of UGS, we are not limiting these to the traditional spaces of urban parks but rather, as Pincetl and Gearin (2005) suggest, use this as a broader concept as children also value informal and unmanaged areas for their free play and exploration (Jansson et al., 2016). The possibility of manipulation of environments, both unmanaged and managed, needs to become recognized as part of children's play and met with understanding among managers who must deal with the different perspectives on places among adults and children (Jansson et al., 2016).

Current investments in UGS are not without problems. In this regard it is central to ensure that, when allocating green space to a certain area, the long-term and low-income residents are not displaced. This is a key issue for policymakers as they seek to balance the positive effects of green space allocation and the negative effects of eco-gentrification (Jo Black and Richards, 2020). Eco-gentrification affects mainly those from deprived communities, who then see both their rights to nature, and to housing threatened. In this context, it is worth mentioning the theory of “Just Green Enough” proposed by Curran and Hamilton (2018), which aims to reverse this trend by allowing communities to design their own environmental initiatives, and preventing the expulsion of the most disadvantaged from these re-qualified places.

Public and open green spaces may lead to the opportunity to develop outdoor education programs, which have been shown to have significant effects on learning (Hamilton, 2017), contributing also to the diminution of behavior and socialization problems (Chiumento et al., 2018; Engemann et al., 2019), improving cognitive development (McCormick, 2017), enhancing prosocial behavior (Putra et al., 2020) and reducing the risk of diseases characteristic of urban societies (McCracken et al., 2016; Roslund et al., 2020). More importantly, the planning process can itself be place-based and place-conscious, in a way that values local knowledge and it is done with and for the local community (Villanueva et al., 2016; Lloyd et al., 2018).

## Minding the Gap: Children's Green Infrastructure

As stated before, UGS are vital for promoting safe and healthy urban spaces, especially for children (Vidal et al., 2020a). Nevertheless, it is worth remarking that for a city to be healthy, it must first be fair and inclusive. And to be fair and inclusive, the city needs to be a collective construction of several voices. Kalache and Kickbusch (1997) suggest that the foundations for healthy living are established in the first years of life. Therefore, urban planning aimed at promoting a healthy environment for children brings benefits in the short, medium and long term. However, in general, children have seldom been called to participate in the processes of urban planning and design (Bishop and Corkery, 2017). This, in spite of the above mentioned UNICEF programs as well as evidence from the new sociology of childhood/children's studies that have shown how children are capable of a critical understanding of place and of making good and realistic contributions to urban planning and policy (Cele and van der Burgt, 2015; Jansson et al., 2016; Ataol et al., 2019; Hanssen, 2019; Mansfield et al., 2021).

Given the ineffectiveness of traditional urban planning (Rittel and Webber, 1973; Rakodi, 2001; Campos, 2015;, the design of the cities by children can be a solution for promoting inclusive values (Krishnamurthy, 2019). However, that may not happen if, as stated before, these are planned as restricted and over-structured places. Power of imagination is a crucial skill that UGS should enhance. However, children are less likely to develop their imagination and fantasy within restricted and over-structured places, strictly formatted with more urban furniture than natural elements (Woolley, 2008; Ferret, 2021; Vidal et al., 2021b). For that reason, there is an evident need to develop an integrated solution that enables children to fully explore cities' spaces. It was in this context that ARUP (2017) proposed the concept of “Child Infrastructure” (CI) to refer to a network of spaces, streets, nature and interventions focused on the city capacity to attract children and remain healthy. This concept goes beyond designated spaces for children like playgrounds, defending an expanded infrastructure that is properly integrated into the multifunctional urban fabric. This proposal aims to place children at the heart of urban planning and to improve the everyday lives of cities' younger residents through intervention at the neighborhood level. Based on this concept, the Gehl Institute (2017) proposed ten principles for the implementation of a CI to combine the accessibility of activities for different ages with daily routes on safer, more welcoming and user-friendly streets, in addition to a connection with nature: (i) give visibility to children and caregivers; (ii) promote curiosity: (iii) encourage children to get dirty; (iv) improve spaces close to their homes; (v) encourage playing in the street; (vi) promoting collective responsibility for children; (vii) develop a community co-creation; (viii) work without borders; (ix) monitor to know where to improve; and (x) strengthen the best ideas. Building from this concept, we suggest that CGI may help reducing the risks for children living in cities not only by promoting the development of safe spaces and routes where cars traffic is reduced or non-existent, but also by allowing children to feel comfortable and encourage independent mobility within urban spaces. This can only be achieved by looking at urban nature experiences as well as security through children's perspectives, and preventing the adultcentric bias of current urban planning.

Infrastructure can be understood as a political, technological and discursive technology of state governance (Kooy and Bakker, 2008). However, agreeing with Berlant (2016, p. 393), “Infrastructure is not identical to system or structure, as we currently see them, because infrastructure is defined by the movement or patterning of social form.” In this sense, infrastructure can be seen as a “living mediation of what organizes life: the lifeworld of structure. Roads, bridges, schools, food chains, finance systems, prisons, families, districts, norms all the systems that link ongoing proximity to being in a world-sustaining relation” (Berlant, 2016, p. 393). According to the (European Commission, 2013, p. 3), Green infrastructure is “…a strategically planned network of natural and semi-natural areas with other environmental features designed and managed to deliver a wide range of ecosystem services.” This network is responsible for the delivery of health-related and social benefits, namely through the creation of a sense of community and by reducing social exclusion and isolation. Notwithstanding the importance of this concept, a growing body of evidence has focused on infrastructures as emergent social-ecological-technological systems that link more-than-human agencies with social processes and technological systems (Star and Ruhleder, 1996; Grabowski et al., 2017; Markolf et al., 2018). Considering that infrastructures are “networks that facilitate the flow of goods, people, or ideas and allow for their exchange over space” (Larkin, 2013, p. 328), it is also possible that infrastructure users can redefine what infrastructure is for, i.e., an unfinished process that allows a co-creation interaction among children, adults and institutions. This aspect can also be related to the idea of “civic infrastructure” as key to the goal of making urban planning a truly inclusive and democratic process. Civic infrastructure can be understood as “formal and informal institutional as well as sociocultural means of connectivity used in knowledge–action collaboration and networking” (Pezzoli, 2018, p. 192). In times of increasing privatization of infrastructure (Viitanen and Kingston, 2014), civic infrastructure is a key notion to put the focus back on community participation and citizenship rights (Perng and Maalsen, 2020). Children's citizenship is particularly undermined by a dominant discursive rationality paradigm of citizen's participation, which also devalues local knowledge (Davies et al., 2012). CGI can benefit from the aggregation of Green and Civic infrastructures through the development of a network that links society to nature in urban areas (Green) through a co-creation and civic participation (Civic) approach (Ito et al., 2016).

The accessibility discussion on UGS is related to the distribution (and availability) of these spaces in the city, but also to mobility, with accessibility being often defined as the availability of green spaces within a short walking distance of the residence area (Boone et al., 2009). However, assessing UGS remains a complex and controversial issue, namely because these spaces are highly heterogeneous in terms of size, design, available structures and services, quality and safety (Wolch et al., 2014). The fact that there are accessible UGS does not mean that they are of quality, safe and suitable for the populations residing in that area, neither, as Iveson (2007) points out, the access to these spaces from a topographical perspective guarantees status as a member of the public, nor the possibility of participating in public actions. Bristol City Council (2008) provided some recommendations regarding the availability of green spaces. The first concerns its quality and represents the main priority. UGS must have the quality to meet the needs of users, where children are included. The second refers to the distance between green spaces and residential/school areas. The third is related to the availability of green spaces in a given geographic area. The relevance of these priorities is based on the principle that quantity should not be the main criterion. Quality and accessibility (distance) are the main priorities to promote democratization in access to green spaces. And the evaluation of these two criteria has to be done with and for children.

**Figure 1:**
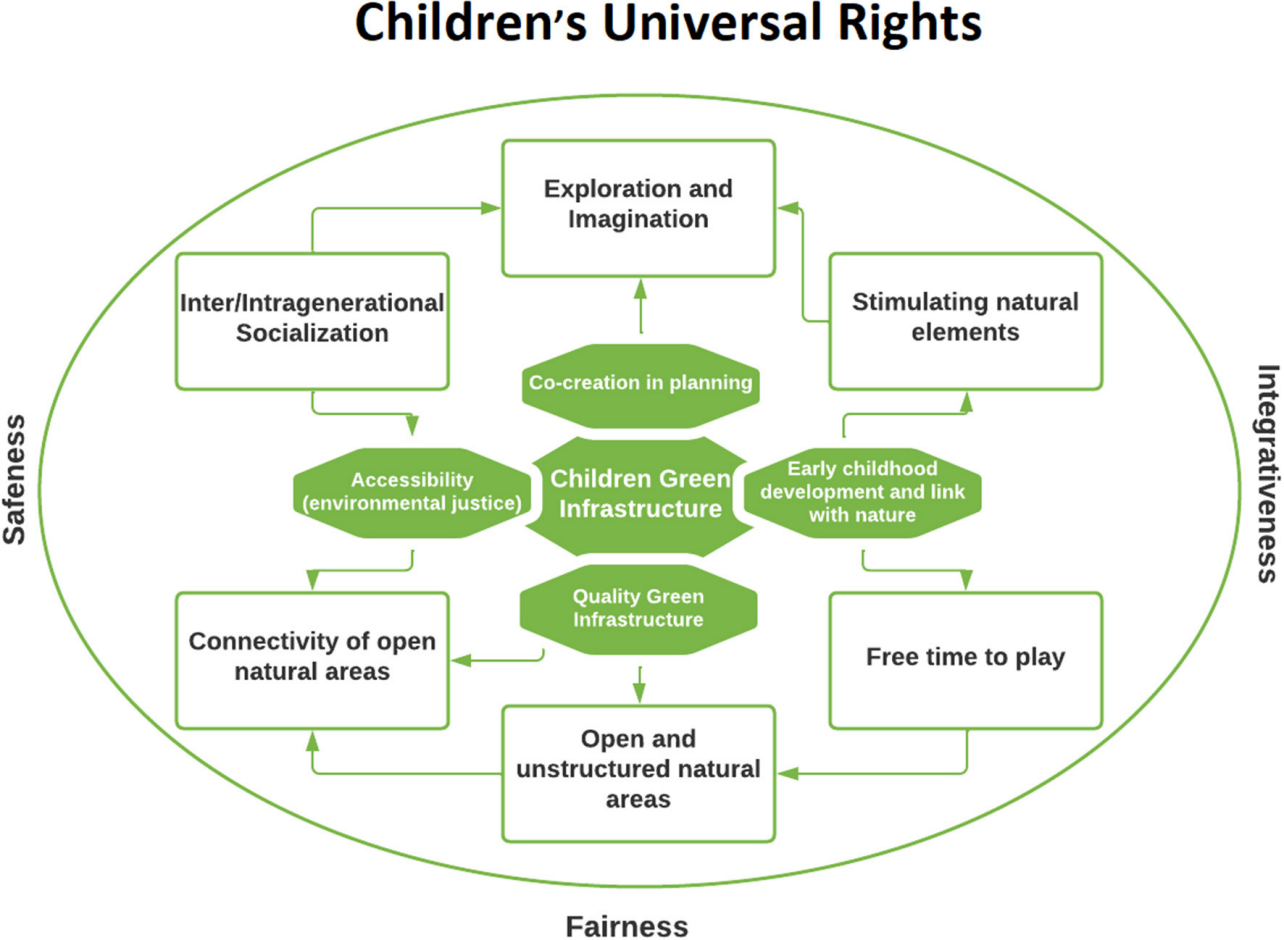
Children green infrastructure model proposed by the authors.

Despite the importance of the CI concept and the literature on the UGS and their accessibility, as presented above, there is an urgent need for a cross-cutting dialogue between these perspectives and the CFC experiences. It is with this aim in mind that we propose the concept of CGI (Figure 1). In the center of the model are framed the main pillars that support CGI. Thus, the process of co-creation should be the basis for creating quality and accessible UGS that promote children's connectivity to nature from early childhood. Around them, are the main objectives that can be achieved through the four pillars, which are interconnected and mutually influenced. By developing stimulating natural elements and fostering intra and inter-generational socialization CGI should promote space exploration and imagination skills. CGI should also contribute to the development of open and unstructured natural areas, and their inter-connectivity. But for CGI to be effective, children must also be provided more free time to play and the possibility to play outdoors. In the larger external circle are the pivotal principles that guide CGI, namely the close link it holds with Children's Rights that frames it. Key to CGI are also the principles of inclusiveness, integrativeness and fairness (of both the urban planning process and the green spaces that are created) and safeness of these green spaces. These have to be understood from the perspective of the children as to prevent adultism bias, but their meaning varies according to the context, as explained before.

CGI is suggested here as an open concept, giving its users, namely children, the possibility to redefine their meaning and elements, within the framework of children's rights. Although this concept derives from the CI concept, it places the idea of connecting children to nature at the center, focusing on the context where they live, learn and play. Looking back, over the past years, due to the intense urbanization process, increased criminality and changing lifestyles and habits, childhood has moved indoors, generating a disconnection from the natural world and a nature-deficit disorder (Phenice and Griffore, 2003; Louv, 2005; Karsten and Felder, 2015; Tsevreni, 2015; Sarmento, 2018). Nonetheless, a new social dynamic is gaining expression related to the gentrification phenomenon, where middle class families are beginning to reclaim city centers (Lilius, 2019), namely the 'YUPPs'—young, urban professional parents—who are actively choosing to live in city centers, rather than taking the traditional route of moving out to the suburbs as soon as they have children” (Karsten, 2014, p. 14). This process has, on the other hand, led to increased practices of “consuming the city” by children and their families (Karsten and Felder, 2015), and cannot be dissociated from the commodification and privatization of urban public spaces. The latter processes have transformed urban public places as well as children's relationship with the city and urban nature since those living in cities, report fewer nature interactions compared with rural counterparts (Collado et al., 2015).

CGI proposal assumes that when cities become places of nearby nature connection, children, families and the environment thrive. CGI is embodied by the Convention on the Rights of the Child (United Nations General Assembly, 1989). It thus recognizes children's rights to freedom of expression, to be heard and to participate in the decisions that affect them. It also builds from the literature briefly presented in this paper and concerning children's rights to play and to nature as well as their right to the city. However, the inclusion of children in urban planning is a complex task that should be realized through a co-creation approach. This becomes more obvious when current evidence shows that children without nature exposure experiences may have little empathy with or interest in nature (Kaplan, 1989). Despite this, and as previously stated; “most children have a natural affinity with nature” (Gill, 2011, p. 8). This means that even though some children have less empathy with nature, if stimulated, they can develop a deeper connection with it. Also, Freeman et al. (2015) argue that the concept of “nature deficit disorder” proposed by Louv (2005) “is adult-determined, and ignores the richness of biodiversity and associated nature connection opportunities available to children within many urban landscapes.” Instead, the authors argue for “a more positive interpretation” in order to explore “whether children's urban lives are and can be nature-rich”, also because what may appear to an adult to be a nature-deficit may in fact be nature-rich from the child's perspective (Louv, 2005, p. 179).

CGI can act in both ways: firstly, by integrating children's preferences and motivations regarding natural elements in city planning and design, but also their own experiences and perceptions and how they interact with nature in their immediate environment. Secondly, through this integration of children's perspectives, it will be possible to create stimulating and more diverse open nature areas interconnected in cities, benefiting particularly children with poor access to formal UGS. Therefore, CGI is here understood as a place-based community-focused continuous and unfinished process whereby both adults and children can participate. What is important is to ensure that children (and other members of society) have the possibility to influence the final outcome through a democratic /participatory and co-design process, meaning that their voices are not only heard but taken into consideration in the final decisions, meeting children's right to participation as enshrined in article 12 of the Convention on the Rights of the Child (CRC).

At the center of CGI is the need to rewild cities, through a co-creation process where children are included and their voices, needs and rights are taken into consideration. The benefits of rewilding cities go far beyond the potential to promote biodiversity recovery and sustainable development. Due to their informal and unrestricted character, rewilding cities provides stimulating natural elements which enhance the exploration and imagination skills of children (Henderson and Vikander, 2008; Gurholt and Sanderud, 2016; Bento and Dias, 2017). Also, rewilding means that nature takes its course and wildlife can flourish (Xie and Bulkeley, 2020; Lehmann, 2021). This solution may lead the opportunity to reintroduce lost biodiversity back into cities spaces and promote inter and intragenerational communication and a closer connection with nature, goals that can be associated with the Planetary Boundaries proposed by Steffen et al. (2015), and its application to city level proposed by Hoornweg et al. (2016). Planetary boundaries cannot be directly applied at a local level, but an effort should be made since cities are key drivers and those most impacted by global influences. Rewilding allows for connecting small and medium natural areas into a continuum, limiting the negative effects of fragmentation, minimizing physical and symbolic distance for children to these areas. Also, CGI may help to address biodiversity loss and extinction through the creation of new habitats to plants and animals.

CGI aims to place nature where children are present, in an equitable manner. Processes of neoliberal urbanization have increased urban inequality regarding resources' distribution, including nature and UGS provision (Authier and Lehman-Frisch, 2013; Lang and Rothenberg, 2017; Karsten, 2020). Since structural deficiencies are harsh to deal with, policymakers and stakeholders must use the power of imagination and creativity to add quality and child-friendly natural features where is possible. If children spend most of the time at school, this process must include transforming schoolyards into green areas or providing nature play options close to childhood centers, adapting vacant lots and streetscapes and opening community gardens. However, it is also important to try to reduce the duration of classes and the amount of homework that children are given and which consumes most of their free time.

More difficult is to establish green corridors aiming to, first, connect children daily routes—home, school, extracurricular activities—and, second, connect the available natural areas in the city. In order for children to walk or bike to school, one needs to create an infrastructure that enables them to do this route in a safe, interesting and playful way, which implies avoiding the conflict with automobile traffic (Becker et al., 2018). In fact, and according to several authors, the increased mobility for adults, namely by the use of the car, resulted in the reduction of children's mobility (Parr, 1967; Engwicht, 1992; Tranter and Sharpe, 2008). In this line, Cervero et al. (2017) developed the concept of kid-friendly Transit-oriented developments (TOD), arguing that planning cities should have the needs of children in mind. Kid-friendly TOD comprise lush and green communal gardens, playgrounds, tot-lots, and play-inviting open space, where surface parking is progressively replaced by gardens and play areas. But this is not enough, as we also need to change adults' minds so that they let their children walk or bike to school, because even if the routes or the infrastructures are safe, their representation may not be. Hence, we have to look at the infrastructure as not only a material thing, but as an imagined and socially constructed place in a world where adults have power over children and their mobility.

The model here proposed through CGI rejects the KFC design (Kit, Fence, Carpet) (Woolley and Lowe, 2013), or even the presence of just one of these elements—with an emphasis on the fences and a standardized children's equipment kit, which is neither flexible nor very stimulating for the children (Castro Seixas et al., 2022). Unstructured open and natural areas can stimulate children's sociospatial exploration and imagination and as Duhn et al. (2017) say, help “troubling the intersections of nature/urban/childhood” (p. 1358).

Inclusiveness is another key aspect of CGI. Our understanding of inclusiveness goes beyond the age variable to ensure the participation of children who are systematically excluded and not just those who have reduced mobility, who have already attracted some attention from designers. We should focus also on including street children, children from poor and stigmatized neighborhoods, institutionalized children, children with visual, hearing, cognitive disabilities and children with mental disorders. This is of utmost importance since children are also victims of social crimes and, poorly lighted “wild green spaces” could present a risk in this regard (Lyytimäki et al., 2008; Matzopoulos et al., 2020). CGI can help to minimize this risk by creating a dynamic and interconnected network through a green infrastructure. As Wenger et al. (2021) state, inclusion is a complex process that involves an interaction between physical (related to the UGS design), social (related to norms and attitudes) and political (related to options and regulations) dimensions. Social class, ethnicity, skin color, racial and gender identity are other important variables to be considered, which leads us to an analysis of how these open and green spaces can constitute a microcosm of society power relations. Indeed, there is some evidence that different social and ethnic groups value different characteristics of UGS and also use the space differently (Loukaitou-Sideris and Sideris, 2010; Özgüner, 2011; Vidal et al., 2022).

CGI also calls for intra and intergenerational socialization among children, young people and adults as well as between children and non-human species. The inclusiveness and integrativeness of CGI could be somewhat related to the concept of Intergenerational Contact Zones (Kaplan et al., 2020), which appeals to a co-creation process among all generations. Azevedo's proposal (Azevedo, 2020) of an intergenerational space can be easily adapted to the development of CGI, in accordance also to a place-based and place-conscious perspective that values local knowledge and community participation (Malone, 2016; Villanueva et al., 2016), including the knowledge and participation of children and young people. In the case of CGI, it is believed that cocreation between communities and decision-makers (Sanders and Stappers, 2008; Lund, 2018; Šuklje Erjavec and Ruchinskaya, 2019; Costa et al., 2020) may better promote children's participation in urban planning and design process. Furthermore, and of utmost importance, community involvement goes far beyond the creation of CGI. As a continuous unfinished process, the success of CGI implies the commitment of the whole community to assure the sustainability of the initiative. CGI calls for a universal commitment of all adults and institutions for the inclusion of children in urban planning processes.

## Final Remarks

In this paper we have built from the literature on UGS, Child-Friendly Cities and environments and Children's Infrastructure to propose the new concept of Children Green Infrastructure (CGI). Our conceptual model is framed upon Children's Rights, although we also recognize a need to critically review official conceptualizations of children's rights as enshrined in the CRC.

We have presented CGI as anchored on several principles that have been highlighted by the literature here reviewed, namely the principle of inclusiveness, which foregrounds the need the include children in urban planning processes, and among these, the most marginalized and excluded children, and the principles of safety, accessibility and quality of the green infrastructures, understanding these features from children's perspectives. This child-centered approach should thus prevent current urban planning adult-centrism and promote children's rights of participation.

As stated before, CGI aims to promote children's rights of participation, to nature and to play. Nevertheless, we believe this model could have beneficial social, environmental and health outcomes for all, namely by helping to rewild cities, providing quality, accessible and child-friendly green infrastructures and fostering inter-generational dialogue and socialization. Indeed, rewilding cities and enhancing biodiversity are essential processes for the promotion of CGI. This perspective implies a different approach than the one inspiring the creation of designated spaces for children, which tend to be highly structured, restricted and regulated, conditioning the exploration of space and the free play of children, especially for the most vulnerable ones (Lehmann, 2021). CGI, through its civic and participatory focus, aims to co-create natural areas by rewilding cities and creating open natural spaces that can be appropriated by children as their own, because children also participate in their design, together with adults and young people. Rewild cities is also a process to reintroduce nature which provides a wide range of benefits to tackle the biodiversity and climate crises in a balanced way that fosters humans and wildlife needs to create better urban landscapes for all.

Access to quality and child-friendly green infrastructures is unequally distributed. The concept here proposed of CGI should be translated in the mission to reconnect children with nature from where they live, learn and play. This implies including the diversity of children's voices in the urban planning process, to create healthier and more inclusive and resilient cities for all to live, grow up in, and realize their full potential. CGI appeals to the need to shape city planning and urban design around children's needs but also their rights, including their right to nature, their right to play and their right to place-making and to participation in urban policies. At the same time though, CGI aims to develop spaces and design processes that promote inter-generational dialogue and socialization, that recognize children as social and political subjects and knowledge producers, capable of contributing to urban design processes together with adults. The green infrastructures created through this approach should also be inter-generational spaces that do not separate children from adults.

Finally, the proposed model should also be considered as a motivation to pursue further research and policy development. In this regard, there are many under-explored areas, such as investigating children's—and especially marginalized children's perspectives and experiences with urban nature; developing child-friendly participation processes and developing methods for fostering inter-generational dialogue, as well as promoting effective dialogue between lay people, experts and policy-makers within urban planning processes. The meaning of the aspects highlighted in the model will be different according to the geographical, historical, cultural and socioeconomic contexts and these aspects also need further analysis. The relevance of some of these aspects may speaks more to the context of post-industrial neoliberal cities of the “Global North”, where the need for promoting children's connection with nature from early childhood as well as children's free time to play and to do so in open and unstructured natural areas is more significantly felt. Nonetheless, questions of deep social inequalities, child's labor and child's participation in armed conflicts, more salient in the “Global South” also prevent children's right, and available time to play, by threatening the very right to childhood. Although children's right to the city, to nature and to play relate to different questions and have different expressions according to the context, we believe the value of CGI remains universal.

## Data Availability Statement

The original contributions presented in the study are included in the article/supplementary material, further inquiries can be directed to the corresponding author/s.

## Author Contributions

DV and EC contributed to conception and design of the study. DV wrote the first draft of the manuscript. EC wrote sections of the manuscript. All authors have contributed to manuscript's final version, read, and approved the submitted version.

## Funding

This work was supported by FCT, I.P., the Portuguese National Funding Agency for Science, Research and Technology, under the Project PTDC/SOC-SOC/30415/2017.

## Conflict of Interest

The authors declare that the research was conducted in the absence of any commercial or financial relationships that could be construed as a potential conflict of interest.

## Publisher's Note

All claims expressed in this article are solely those of the authors and do not necessarily represent those of their affiliated organizations, or those of the publisher, the editors and the reviewers. Any product that may be evaluated in this article, or claim that may be made by its manufacturer, is not guaranteed or endorsed by the publisher.

## Footnotes

^1^The studies cited in this paragraph do not use, nor build from the UGS concept. In fact, there seems to be two different perspectives in terms of the literature with relevance to this topic: on the one hand, the interdisciplinary studies that build specifically from the UGS concept, and on the other hand, the new children's studies that have given an important contribution to the understanding of children's relationship with urban places and urban nature, and the recognition of children as social and political actors, knowledge-producers and subjects of rights. Our proposal of the Child Green Infrastructure concept builds from the articulation between these two perspectives.

## References

[B1] Abercrombie L. C. Sallis J. F. Conway T. L. Frank L. D. Saelens B. E. Chapman J. E. (2008). Income and racial disparities in access to public parks and private recreation facilities. Am. J. Prev. Med. 34, 9–15. 10.1016/j.amepre.2007.09.030 18083445

[B2] Antonovsky A. (1979). Health, Stress, and Coping. San Francisco: Jossey-Bass.

[B3] ARUP (2017). Cities Alive: Designing for Urban Childhoods. Londres: ARUP.

[B4] Ataol Ö. Krishnamurthy S. van Wesemael P. (2019). Children's participation in urban planning and design: a systematic review. Child. Youth Environ. 29, 27. 10.7721/chilyoutenvi.29.2.0027

[B5] Authier J. Y. Lehman-Frisch S. (2013). Le Goût des Autres: Gentrification Told by Children. Urban Stud. 50, 994–1010. 10.1177/0042098012465127

[B6] Azevedo C. (2020). “Urban Public Parks,” in Intergenerational Contact Zones: Place-based Strategies for Promoting Social Inclusion and Belonging, editors M. Kaplan, L. L. Thang, M. Sánchez, and J. Hoffman (London: Routledge).

[B7] Becker A. Lampe S. Negussie L. Schmal P. C. (2018). Ride a Bike!: Reclaim the City. Basel: Birkhauser.

[B8] Bensalah L. Caillies S. Anduze M. (2016). Links among cognitive empathy, theory of mind, and affective perspective taking by young children. J. Genet. Psychol. 177, 17–31. 10.1080/00221325.2015.1106438 26508454

[B9] Bento G. Dias G. (2017). The importance of outdoor play for young children's healthy development. Porto Biomed. J. 2, 157–160. 10.1016/j.pbj.2017.03.003 32258612 PMC6806863

[B10] Berlant L. (2016). The commons: infrastructures for troubling times^*^. Environ. Plan. D Soc. Sp. 34, 393–419. 10.1177/0263775816645989

[B11] Bishop K. Corkery L. (2017). Designing Cities With Children and Young People: Beyond Playgrounds and Skate Parks. Nova Iorque: Routledge.

[B12] Boone C. G. Buckley G. L. Grove J. M. Sister C. (2009). Parks and people: an environmental justice inquiry in Baltimore, Maryland. Ann. Assoc. Am. Geogr. 99, 767–787. 10.1080/00045600903102949

[B13] Bristol City Council (2008). Bristol's Parks and Green Space Strategy. Bristol: Visual Technology. Available online at: http://scholar.google.com/scholar?hl=en&btnG=Search&q=intitle:Bristol?s+Parks+and+Green+Space+Strategy#0%5Cn http://scholar.google.com/scholar?hl=en&btnG=Search&q=intitle:Bristol's+parks+and+green+space+strategy%230 (accessed September 19, 2021).

[B14] Brown C. de Lannoy A. McCracken D. Gill T. Grant M. Wright H.. (2019). Special issue: child-friendly cities. Cities Heal. 3, 1–7. 10.1080/23748834.2019.1682836

[B15] Campos V. Ferrão J. (2015). O Ordenamento do território: uma perspetiva genealógica. ICS Work. Pap. 1, 1–45.

[B16] Castro Seixas E. Giacchetta N. (2020). “Direito das crianças à cidade e resiliência urbana em tempos de Covid-19,” in Crianças na cidade em tempos de Covid-19: Reflexões a partir da investigação em espaços públicos no Porto e em Lisboa. Cadernos da Pandemia. Vol. 6, editor E. Castro Seixas (Porto: Instituto de Sociologia da Universidade do Porto), 26–33. Available online at: https://repositorio-aberto.up.pt/bitstream/10216/131360/2/435046.pdf (accessed September 20, 2021).

[B17] Castro Seixas E. Tomás C. Giachetta N. (2022). “A Produção Social da Infância nos Parques Urbanos de Lisboa,” in O direito das crianças à cidade: perspectivas desde o Brasil e Portugal, editirs M. A. Gobbi, C. I. dos Anjos, E. C. Seixas, and C. Tomás (São Paulo: Universidade de São Paulo).

[B18] Cele S. van der Burgt D. (2015). Participation, consultation, confusion: professionals' understandings of children's participation in physical planning. Child. Geogr. 13, 14–29. 10.1080/14733285.2013.827873

[B19] Cervero R. Guerra E. Al S. (2017). Beyond Mobility: Planning Cities for People and Places. Washington, D.C.: Island Press.

[B20] Chiumento A. Mukherjee I. Chandna J. Dutton C. Rahman A. Bristow K. (2018). A haven of green space: learning from a pilot pre-post evaluation of a school-based social and therapeutic horticulture intervention with children. BMC Public Health 18, 836. 10.1186/s12889-018-5661-9 29976193 PMC6034303

[B21] Collado S. Corraliza J. A. Staats H. Ruíz M. (2015). Effect of frequency and mode of contact with nature on children's self-reported ecological behaviors. J. Environ. Psychol. 41, 65–73. 10.1016/j.jenvp.2014.11.001

[B22] Cordero Arce M. (2012). Towards an emancipatory discourse of children's rights. Int. J. Child. Rights 20, 365–421. 10.1163/157181812X637127

[B23] Costa C. S. Mačiuliene M. Menezes M. Marušić B. G. (2020). Co-creation of Public Open Places. Practice - Reflection - Learning. Lisboa: Edições Universitárias Lusófonas.

[B24] Curran W. Hamilton T. (2018). Just Green Enough: Urban Development and Environmental Gentrification. London: Routledge.

[B25] Dadvand P. Gascon M. Markevych I. (2019). “Green spaces and child health and development,” in Biodiversity and Health in the Face of Climate Change, editors M. R. Marselle, J. Stadler, H. Korn, K. N. Irvine, and A. Bonn (Cham: Springer International Publishing), 121–130.

[B26] Davey C. Lundy L. (2011). Towards greater recognition of the right to play: an analysis of article 31 of the UNCRC. Child. Soc. 25, 3–14. 10.1111/j.1099-0860.2009.00256.x

[B27] Davies S. R. Selin C. Gano G. Pereira Â. G. (2012). Citizen engagement and urban change: three case studies of material deliberation. Cities 29, 351–357. 10.1016/j.cities.2011.11.012

[B28] DeLoache J. S. Pickard M. B. LoBue V. (2010). “How very young children think about animals,” in How Animals Affect Us: Examining the Influences of Human–Animal Interaction on Child Development and Human Health, editors P. McCardle, S. McCune, J. A. Griffin, and V. Maholmes (Washington, D.C.: American Psychological Association), 85–99.

[B29] Derr V. Corona Y. Gülgönen T. (2019). Children's perceptions of and engagement in urban resilience in the United States and Mexico. J. Plan. Educ. Res. 39, 7–17. 10.1177/0739456X17723436

[B30] Duhn I. Malone K. Tesar M. (2017). Troubling the intersections of urban/nature/childhood in environmental education. Environ. Educ. Res. 23, 1357–1368. 10.1080/13504622.2017.1390884

[B31] Engemann K. Pedersen C. B. Arge L. Tsirogiannis C. Mortensen P. B. Svenning J. C. (2018). Childhood exposure to green space – A novel risk-decreasing mechanism for schizophrenia? Schizophr. Res. 199, 142–148. 10.1016/j.schres.2018.03.026 29573946

[B32] Engemann K. Pedersen C. B. Arge L. Tsirogiannis C. Mortensen P. B. Svenning J. C. (2019). Residential green space in childhood is associated with lower risk of psychiatric disorders from adolescence into adulthood. Proc. Natl. Acad. Sci. U. S. A. 116, 5188–5193. 10.1073/pnas.1807504116 30804178 PMC6421415

[B33] Engwicht D. (1992). Towards an Eco-City: Calming the Traffic. Sydney: Envirobook.

[B34] European Commission (2013). Green Infrastructure (GI) — Enhancing Europe's Natural Capital - COM(2013) 149. Brussels: European Union. Available online at: https://eur-lex.europa.eu/legal-content/EN/ALL/?uri=CELEX:52013DC0249 (accessed September 20, 2021).

[B35] Faber Taylor A. Kuo F. E. (2009). Children with attention deficits concentrate better after walk in the park. J. Atten. Disord. 12, 402–409. 10.1177/1087054708323000 18725656

[B36] Ferret M. P. (2021). Childhood, nature and lock-down. Finisterra Rev. Port. Geogr. 55, 169–174. 10.18055/Finis20352

[B37] Fjørtoft I. (2004). Landscape as playscape: the effects of natural environments on children's play and motor development. Child. Youth Environ. 14, 21–44.

[B38] Fjørtoft I. Sudo T. Ito K. (2021). “Nature in the cities: places for play and learning,” in Urban Biodiversity and Ecological Design for Sustainable Cities, editor K. Ito (Tokyo: Springer), 125–141.

[B39] Formoso D. Weber R. N. Atkins M. S. (2010). Gentrification and urban children's well-being: tipping the scales from problems to promise. Am. J. Community Psychol. 46, 395–412. 10.1007/s10464-010-9348-3 20941538

[B40] Freeman C. Heezik Y. van Hand K. Stein A. (2015). Making cities more child- and nature-friendly: a child-focused study of nature connectedness in New Zealand Cities. Child. Youth Environ. 25, 176. 10.7721/chilyoutenvi.25.2.0176

[B41] Gehl Institute (2017). Space to Grow: Ten Principles That Support Happy, Healthy Families in a Playful, Friendly City. Copenhagen. Available online at: https://gehlinstitute.org/wp-content/uploads/2018/04/GehlInstitute_SpaceToGrow_single_pages.pdf (accessed September 15, 2021).

[B42] Gill T. (2007). No Fear: Growing Up in a Risk Society. Lisboa: Fundação Calouste Gulbenkian.

[B43] Gill T. (2011). Sowing the Seeds Reconnecting London's Children with Nature. London: Greater London Authority.

[B44] Gillett-Swan J. Thelander N. (2021). Children's Rights From International Educational Perspectives. Cham: Springer.

[B45] Grabowski Z. J. Matsler A. M. Thiel C. McPhillips L. Hum R. Bradshaw A.. (2017). Infrastructures as socio-eco-technical systems: five considerations for interdisciplinary dialogue. J. Infrastruct. Syst. 23, 02517002. 10.1061/(ASCE)IS.1943-555X.0000383

[B46] Graça M. Alves P. Gonçalves J. Nowak D. J. Hoehn R. Farinha-Marques P.. (2018). Assessing how green space types affect ecosystem services delivery in Porto, Portugal. Landsc. Urban Plan. 170, 195–208. 10.1016/j.landurbplan.2017.10.007

[B47] Gurholt K. P. Sanderud J. R. (2016). Curious play: children's exploration of nature. J. Adventure Educ. Outdoor Learn. 16, 318–329. 10.1080/14729679.2016.1162183

[B48] Haikkola L. Pacilli M. G. Horelli L. Prezza M. (2007). Interpretations of urban child-friendliness: a comparative study of two neighborhoods in Helsinki and Rome. Child. Youth Environ. 17, 319–351.

[B49] Haines-Young R. Potschin M. B. (2018). Common International Classification of Ecosystem Services (CICES) V5.1 and Guidance on the Application of the Revised Structure. Nottingham. Available online at: www.cices.eu (accessed September 10, 2021).

[B50] Hamilton J. M. (2017). Relationships Between Outdoor and Classroom Task Settings and Cognition in Primary Schoolchildren. Available online at: http://hdl.handle.net/10399/3253 (accessed October 2, 2021).

[B51] Hand K. L. Freeman C. Seddon P. J. Recio M. R. Stein A. Van Heezik Y. (2017). The importance of urban gardens in supporting children's biophilia. Proc. Natl. Acad. Sci. U. S. A. 114, 274–279. 10.1073/pnas.1609588114 28028204 PMC5240683

[B52] Hanssen G. S. (2019). The social sustainable city: how to involve children in designing and planning for urban childhoods? Urban Plan. 4, 53–66. 10.17645/up.v4i1.1719

[B53] Henderson B. Vikander N. (2008). Nature First: Outdoor Life the Friluftsliv Way. Toronto: Heritage Books.

[B54] Hoffimann E. Barros H. Ribeiro A. I. (2017). Socioeconomic inequalities in green space quality and accessibility—evidence from a Southern European city. Int. J. Environ. Res. Public Health. 14, 916. 10.3390/ijerph14080916 28809798 PMC5580619

[B55] Hoornweg D. Hosseini M. Kennedy C. Behdadi A. (2016). An urban approach to planetary boundaries. Ambio 45, 567–580. 10.1007/s13280-016-0764-y 26897006 PMC4980311

[B56] Ito K. (2021). Urban Biodiversity and Ecological Design for Sustainable Cities. Tokyo: Springer.

[B57] Ito K. Sudo T. Fjørtoft I. (2016). “Ecological design: collaborative landscape design with school children,” in Children, Nature, Cities, editors A. M. Murnahan and L. J. Shillington (London and New York: Routledge, Taylor & Francis Group), 195–209.

[B58] Iveson K. (2007). Publics and the City. Oxford and Malden: Blackwell.

[B59] Jansson M. Sundevall E. Wales M. (2016). The role of green spaces and their management in a child-friendly urban village. Urban For. Urban Green. 18, 228–236. 10.1016/j.ufug.2016.06.014

[B60] Jennings V. Bamkole O. (2019). The relationship between social cohesion and urban green space: an avenue for health promotion. Int. J. Environ. Res. Public Health 16, 452. 10.3390/ijerph16030452 30720732 PMC6388234

[B61] Jennings V. Larson L. Yun J. (2016). Advancing sustainability through urban green space: cultural ecosystem services, equity, and social determinants of health. Int. J. Environ. Res. Public Health 13, 196. 10.3390/ijerph13020196 26861365 PMC4772216

[B62] Jennings V. Reid C. E. Fuller C. H. (2021). Green infrastructure can limit but not solve air pollution injustice. Nat. Commun. 12, 4681. 10.1038/s41467-021-24892-1 34344872 PMC8333325

[B63] Jo Black K. Richards M. (2020). Eco-gentrification and who benefits from urban green amenities: NYC's high line. Landsc. Urban Plan. 204, 103900. 10.1016/j.landurbplan.2020.103900

[B64] Johnson-Gaither C. (2011). Latino park access: examining environmental equity in a new destination county in the South. J. Park Recreat. Admi. 29, 37–52.

[B65] Kabisch N. Strohbach M. Haase D. Kronenberg J. (2016). Urban green space availability in European cities. Ecol. Indic. 70, 586–596. 10.1016/j.ecolind.2016.02.029

[B66] Kahn P. H. (1997). Developmental psychology and the biophilia hypothesis: children's affiliation with nature. Dev. Rev. 17, 1–61. 10.1006/drev.1996.0430

[B67] Kalache A. Kickbusch I. (1997). A global strategy for healthy ageing. World Health 50, 4–5.

[B68] Kaplan M. Thang L. L. Sánchez M. Hoffman J. (2020). Intergenerational Contact Zones: Place-Based Strategies for Promoting Social Inclusion and Belonging. London: Routledge.

[B69] Kaplan R. Kaplan S. (1989). The Experience of Nature: A Psychological Perspective. New York, NY: Cambridge University Press.

[B70] Karsten L. (2007). Housing as a way of life: Towards an understanding of middle-class families' preference for an urban residential location. Hous. Stud. 22, 83–98. 10.1080/02673030601024630

[B71] Karsten L. (2014). “Families are beginning to reclaim city centres,” in Early Child. Matters, 14–16. Available online at: https://earlychildhoodmatters.online/wp-content/uploads/2019/06/ECM123-2014_Small-children_big-cities.pdf (accessed September 25, 2021).

[B72] Karsten L. (2020). Counterurbanisation: why settled families move out of the city again. J. Hous. Built Environ. 35, 429–442. 10.1007/s10901-020-09739-3

[B73] Karsten L. Felder N. (2015). Parents and children consuming the city: geographies of family outings across class. Ann. Leis. Res. 18, 205–218. 10.1080/11745398.2015.1011679

[B74] Kazemi F. Abolhassani L. Rahmati E. A. Sayyad-Amin P. (2018). Strategic planning for cultivation of fruit trees and shrubs in urban landscapes using the SWOT method: a case study for the city of Mashhad, Iran. Land Use policy 70, 1–9. 10.1016/j.landusepol.2017.10.006

[B75] Keith R. J. Given L. M. Martin J. M. Hochuli D. F. (2021). Urban children's connections to nature and environmental behaviors differ with age and gender. PLoS ONE 16, e0255421. 10.1371/journal.pone.0255421 34324598 PMC8321113

[B76] Kleinschroth F. Kowarik I. (2020). COVID-19 crisis demonstrates the urgent need for urban greenspaces. Front. Ecol. Environ. 18, 318–319. 10.1002/fee.2230 32834788 PMC7436739

[B77] Kooy M. Bakker K. (2008). Technologies of government: constituting subjectivities, spaces, and infrastructures in colonial and contemporary Jakarta. Int. J. Urban Reg. Res. 32, 375–391. 10.1111/j.1468-2427.2008.00791.x

[B78] Krishnamurthy S. (2019). Reclaiming spaces: child inclusive urban design. Cities Heal. 3, 86–98. 10.1080/23748834.2019.1586327

[B79] Kyttä M. (2004). The extent of children's independent mobility and the number of actualized affordances as criteria for child-friendly environments. J. Environ. Psychol. 24, 179–198. 10.1016/S0272-4944(03)00073-2

[B80] Lang S. Rothenberg J. (2017). Neoliberal urbanism, public space, and the greening of the growth machine: New York City's High Line park. Environ. Plan. A 49, 1743–1761. 10.1177/0308518X16677969

[B81] Larkin B. (2013). The politics and poetics of infrastructure. Annu. Rev. Anthropol. 42, 327–343. 10.1146/annurev-anthro-092412-155522

[B82] Laurent É. (2011). Issues in environmental justice within the European Union. Ecol. Econ. 70, 1846–1853. 10.1016/j.ecolecon.2011.06.02535096181

[B83] Lefebvre H. (1974). La production de l'espace. Paris: Anthropos.

[B84] LeFrançois B. A. (2014). “Adultism,” in Encyclopedia of Critical Psychology, editor T. Teo (New York, NY: Springer), 517–523.

[B85] Lehmann S. (2021). Growing biodiverse urban futures: renaturalization and rewilding as strategies to strengthen urban resilience. Sustainability 13, 2932. 10.3390/su13052932

[B86] Lencastre M. P. A. Farinha Marques P. (2021). Da Biofilia à Ecoterapia. A Importância dos Parques Urbanos para a Saúde Mental. Trab. Antropol. e Etnol. 61, 131–155.

[B87] Leverett S. (2011). “Children's spaces,” in Children and Young People's Spaces: Developing Practice, editors P. Foley and S. Leverett (Houndsmills: Palgrave Macmillan), 9–24.

[B88] Lilius J. (2019). Reclaiming Cities as Spaces of Middle Class Parenthood. London: Palgrave Macmillan.

[B89] Liotta C. Kervinio Y. Levrel H. Tardieu L. (2020). Planning for environmental justice - reducing well-being inequalities through urban greening. Environ. Sci. Policy 112, 47–60. 10.1016/j.envsci.2020.03.017

[B90] Lloyd A. Truong S. Gray T. (2018). Place-based outdoor learning: more than a drag and drop approach. J. Outdoor Environ. Educ. 21, 45–60. 10.1007/s42322-017-0002-5

[B91] Loukaitou-Sideris A. Sideris A. (2010). What brings children to the park? Analysis and measurement of the variables affecting children's use of parks. J. Am. Plan. Assoc. 76, 89–107. 10.1080/01944360903418338

[B92] Louv R. (2005). Last Child in the Woods: Saving Our Children From Nature-Deficit Disorder. Nova Iorque: Algonquin Books.

[B93] Lund D. H. (2018). Co-creation in urban governance: from inclusion to innovation. Scand. J. Public Adm. 22, 27–41.

[B94] Lyytimäki J. Petersen L. K. Normander B. Bezák P. (2008). Nature as a nuisance? Ecosystem services and disservices to urban lifestyle. Environ. Sci. 5, 161–172. 10.1080/15693430802055524

[B95] Malone K. (2016). “Children's place encounters: place-based participatory research to design a child-friendly and sustainable urban development,” in Geographies of Global Issues: Change and Threat, editors N. Ansell, N. Klocker, and T. Skelton (Singapore: Springer), 501–530.

[B96] Mansfield R. G. Batagol B. Raven R. (2021). “Critical agents of change?”: Opportunities and limits to children's participation in urban planning. J. Plan. Lit. 36, 170–186. 10.1177/0885412220988645

[B97] Markolf S. A. Chester M. V. Eisenberg D. A. Iwaniec D. M. Davidson C. I. Zimmerman R.. (2018). Interdependent infrastructure as linked social, ecological, and technological systems (SETSs) to address lock-in and enhance resilience. Earths Futur. 6, 1638–1659. 10.1029/2018EF000926

[B98] Mathey J. Rößler S. Lehmann I. Bräuer A. (2011). “Urban green spaces: potentials and constraints for urban adaptation to climate change,” in Resilient Cities. Local Sustainability, editor K. Otto-Zimmermann (Dordrecht: Springer), 479–485.

[B99] Matthews H. (1995). Living on the edge: children as ‘outsiders. Tijdschr. voor Econimische en Soc. Geogr. 89, 456–466. 10.1111/j.1467-9663.1995.tb01867.x

[B100] Matzopoulos R. Bloch K. Lloyd S. Berens C. Bowman B. Myers J.. (2020). Urban upgrading and levels of interpersonal violence in Cape Town, South Africa: the violence prevention through urban upgrading programme. Soc. Sci. Med. 255, 112978. 10.1016/j.socscimed.2020.112978 32330747

[B101] Mayen Huerta C. Utomo A. (2021). Evaluating the association between urban green spaces and subjective well-being in Mexico city during the COVID-19 pandemic. Heal. Place 70, 102606. 10.1016/j.healthplace.2021.102606 34139612 PMC9760010

[B102] McCormick R. (2017). Does access to green space impact the mental well-being of children: a systematic review. J. Pediatr. Nurs. Nurs. Care Child. Fam. 37, 3–7. 10.1016/j.pedn.2017.08.027 28882650

[B103] McCracken D. S. Allen D. A. Gow A. J. (2016). Associations between urban greenspace and health-related quality of life in children. Prev. Med. Reports 3, 211–221. 10.1016/j.pmedr.2016.01.013 27419017 PMC4929180

[B104] Neaum S. (2010). Child Development for Early Childhood Studies. London: SAGE Publications Limited.

[B105] Neto C. (2020). Libertem as crianças. A urgência de brincar e ser ativo. Lisboa: Contraponto Editores.

[B106] Özgüner H. (2011). Cultural differences in attitudes towards urban parks and green spaces. Landsc. Res. 36, 599–620. 10.1080/01426397.2011.560474

[B107] Parr A. E. (1967). The Child in the City: urbanity and the urban scene. Landsc. Mag. Hum. Geogr. 17, 3–5.

[B108] Perng S. Y. Maalsen S. (2020). Civic infrastructure and the appropriation of the corporate smart city. Ann. Am. Assoc. Geogr. 110, 507–515. 10.1080/24694452.2019.1674629

[B109] Pezzoli K. (2018). Civic infrastructure for neighborhood planning. J. Am. Plan. Assoc. 84, 191–193. 10.1080/01944363.2018.1424559

[B110] Phenice L. A. Griffore R. J. (2003). Young children and the natural world. Contemp. Issues Early Child. 4, 167–171. 10.2304/ciec.2003.4.2.6

[B111] Pincetl S. Gearin E. (2005). The reinvention of public green space. Urban Geogr. 26, 365–384. 10.2747/0272-3638.26.5.365

[B112] Putra I. G. N. E. Astell-Burt T. Cliff D. P. Vella S. A. John E. E. Feng X. (2020). The relationship between green space and prosocial behaviour among children and adolescents: a systematic review. Front. Psychol. 11, 859. 10.3389/fpsyg.2020.00859 32425867 PMC7203527

[B113] Racelis M. Aguirre A. D. M. (2002). Child rights for urban poor children in child friendly Philippine cities: views from the community. Environ. Urban. 14, 97–114. 10.1177/095624780201400208

[B114] Racelis M. Aguirre A. D. M. (2006). Making Philippine Cities Child Friendly: Voices of Children in Poor Communities. New York, NY: UNICEF.

[B115] Rakodi C. (2001). Forget planning, put politics first? Priorities for urban management in developing countries. ITC J. 3, 209–223. 10.1016/S0303-2434(01)85029-7

[B116] Ribeiro A. I. Triguero-Mas M. Jardim Santos C. Gómez-Nieto A. Cole H. Anguelovski I.. (2021). Exposure to nature and mental health outcomes during COVID-19 lockdown. A comparison between Portugal and Spain. Environ. Int. 154, 106664. 10.1016/j.envint.2021.106664 34082237 PMC8162907

[B117] Rittel H. W. J. Webber M. M. (1973). Dilemmas in a general theory of planning. Policy Sci. 4, 155–169. 10.1007/BF01405730

[B118] Rodgers C. (2020). Nourishing and protecting our urban ‘green' space in a post-pandemic world. Environ. Law Rev. 22, 165–169. 10.1177/1461452920934667

[B119] Roslund M. I. Puhakka R. Grönroos M. Nurminen N. Oikarinen S. Gazali A. M.. (2020). Biodiversity intervention enhances immune regulation and health-associated commensal microbiota among daycare children. Sci. Adv. 6, eaba2578. 10.1126/sciadv.aba2578 33055153 PMC7556828

[B120] Sanders E. B.-N. Stappers P. J. (2008). Co-creation and the new landscapes of design. CoDesign 4, 5–18. 10.1080/15710880701875068

[B121] Sarmento M. J. (2018). Infância e cidade: restrições e possibilidades. Educação 41, 232. 10.15448/1981-2582.2018.2.31317

[B122] Sarmento M. J. Tomás C. (2020). A infância é um direito? Sociol. Rev. da Fac. Let. da Univ. do Porto Número Tem, 15–30. 10.21747/08723419/soctem2020a1

[B123] Skelton T. (2007). Children, young people, UNICEF and participation. Child. Geogr. 5, 165–181. 10.1080/14733280601108338

[B124] Star S. L. Ruhleder K. (1996). Steps toward an ecology of infrastructure: design and access for large information spaces. Inf. Syst. Res. 7, 111–134. 10.1287/isre.7.1.111

[B125] Steffen W. Richardson K. Rockström J. Cornell S. E. Fetzer I. Bennett E. M.. (2015). Planetary boundaries: Guiding human development on a changing planet. Science 347, 1259855. 10.1126/science.1259855 25592418

[B126] Šuklje Erjavec I. Ruchinskaya T. (2019). “A spotlight of co-creation and inclusiveness of public open spaces,” in CyberParks - The Interface Between People, Places and Technology, editos C. Smaniotto Costa, I. Šuklje Erjavec, T. Kenna, M. de Lange, K. Ioannidis, G. Maksymiuk, et al. (Cham: Springer), 209–223.

[B127] Sutton S. E. (1996). Weaving a Tapestry of Resistance: The Places Power and Poetry of a Sustainable Society. Westport: Bergin and Garvey.

[B128] Svetlova M. Nichols S. R. Brownell C. A. (2010). Toddlers' prosocial behavior: from instrumental to empathic to altruistic helping. Child Dev. 81, 1814–1827. 10.1111/j.1467-8624.2010.01512.x 21077866 PMC3088085

[B129] Tendais I. Ribeiro A. I. (2020). Espaços verdes urbanos e saúde mental durante o confinamento causado pela COVID-19. Finisterra Rev. Port. Geogr. 55, 183–188. 10.18055/Finis20184

[B130] Thaler R. H. Sunstein C. R. (2008). Nudge: Improving Decisions About Health, Wealth, and Happiness. Nova Iorque: Yale University Press.

[B131] Tomás C. (2007). Paradigmas, imagens e concepções da infância em sociedades mediatizadas. Media J. 11, 119–134.

[B132] Tranter P. J. Sharpe S. (2008). Escaping monstropolis: child-friendly cities, peak oil and monsters, Inc. Child. Geogr. 6, 295–308. 10.1080/14733280802184021

[B133] Tsevreni I. (2015). Children's social and spatial exclusion in the city. The need for an internal look. Int. J. Crit. Pedagog. 6, 149–168.

[B134] UNICEF (2018). Advantage or Paradox? The Challenge for Children and Young People of Growing Up Urban. Nova Iorque: UNICEF.

[B135] United Nations (2015). Transforming Our World: The 2030 Agenda for Sustainable Development. Resolution adopted by the General Assembly on 25 September 2015, A/RES/70/1. Geneva. Available online at: http://www.un.org/en/development/desa/population/migration/generalassembly/docs/globalcompact/A_RES_70_1_E.pdf (accessed September 19, 2021).

[B136] United Nations General Assembly (1989). Convention on the Rights of the Child. New York, NY: United Nations General Assembly. Available online at: https://treaties.un.org/doc/Treaties/1990/09/1990090203-14~AM/Ch_IV_11p.pdf (accessed October 6, 2021).

[B137] van Vliet W. Karsten L. (2015). Child-friendly cities in a globalizing world: different approaches and a typology of children's roles. Child. Youth Environ. 25, 1. 10.7721/chilyoutenvi.25.2.0001

[B138] Vidal D. G. Barros N. Maia R. L. (2020a). “Public and green spaces in the context of sustainable development,” in Sustainable Cities and Communities, Encyclopedia of the UN Sustainable Development Goals, editors W. Leal Filho, A. M. Azul, L. Brandli, P. G. Özuyar, and T. Wall (Cham: Springer Nature Switzerland AG), 479–487. 10.18055/Finis19813

[B139] Vidal D. G. Dias R. C. Oliveira G. M. Dinis M. A. P. Leal Filho W. Fernandes C. O.. (2022). “A review on the cultural ecosystem services provision of urban green spaces: perception, use and health benefits,” in Sustainable Policies and Practices in Energy, Environment and Health Research, editors W. Leal Filho, D. G. Vidal, M. A. P. Dinis, and R. C. Dias (Cham: Springer).

[B140] Vidal D. G. Fernandes C. O. Viterbo L. M. F. Barros N. Maia R. L. (2020b). “Espaços verdes urbanos e saúde mental: uma revisão sistemática da literatura,” in Actas do 13^*o*^ Congresso Nacional de Psicologia da Saúde, editors H. Pereira, S. Monteiro, G. Esgalhado, A. Cunha, and I. Leal (Lisboa: ISPA), 427–436.

[B141] Vidal D. G. Fernandes C. O. Viterbo L. M. F. V. Vilaça H. Barros N. Maia R. L. (2021a). Combining an evaluation grid application to assess ecosystem services of urban green spaces and a socioeconomic spatial analysis. Int. J. Sustain. Dev. World Ecol. 28, 291–302. 10.1080/13504509.2020.1808108

[B142] Vidal D. G. Fernandes C. O. Viterbo L. M. F. Vilaça H. Barros N. Maia R. L. (2021b). Usos e Perceções sobre Jardins e Parques Públicos Urbanos: Resultados Preliminares de um Inquérito na Cidade Do Porto (Portugal). Finisterra Rev. Port. Geogr. 56, 137–157.

[B143] Viitanen J. Kingston R. (2014). Smart cities and green growth: outsourcing democratic and environmental resilience to the global technology sector. Environ. Plan. A 46, 803–819. 10.1068/a46242

[B144] Villanueva K. Badland H. Kvalsvig A. O'Connor M. Christian H. Woolcock G.. (2016). Can the neighborhood built environment make a difference in children's development? Building the research agenda to create evidence for place-based children's policy. Acad. Pediatr. 16, 10–19. 10.1016/j.acap.2015.09.006 26432681

[B145] Vincelot J. (2019). Urban95: a global initiative linking early childhood development and the urban field. Cities Heal. 3, 40–45. 10.1080/23748834.2018.1538178

[B146] Ward J. S. Duncan J. S. Jarden A. Stewart T. (2016). The impact of children's exposure to greenspace on physical activity, cognitive development, emotional wellbeing, and ability to appraise risk. Heal. Place 40, 44–50. 10.1016/j.healthplace.2016.04.015 27179137

[B147] Wenger I. Schulze C. Lundström U. Prellwitz M. (2021). Children's perceptions of playing on inclusive playgrounds: a qualitative study. Scand. J. Occup. Ther. 28, 136–146. 10.1080/11038128.2020.1810768 32857665

[B148] Wilson E. O. (1984). Biophilia. The Human Bond with Other Species. Cambridge: Harvard University Press.

[B149] Wolch J. R. Byrne J. Newell J. P. (2014). Urban green space, public health, and environmental justice: the challenge of making cities ‘just green enough.' Landsc. Urban Plan. 125, 234–244. 10.1016/j.landurbplan.2014.01.017

[B150] Woolley H. (2008). Watch this space! Designing for children's play in public open spaces. Geogr. Compass 2, 495–512. 10.1111/j.1749-8198.2008.00077.x

[B151] Woolley H. Lowe A. (2013). Exploring the relationship between design approach and play value of outdoor play spaces. Landsc. Res. 38, 53–74. 10.1080/01426397.2011.640432

[B152] Wortzel J. D. Wiebe D. J. DiDomenico G. E. Visoki E. South E. Tam V.. (2021). Association between urban greenspace and mental wellbeing during the COVID-19 pandemic in a U.S. Cohort. Front. Sustain. Cities 3, 686159. 10.3389/frsc.2021.686159

[B153] Xie L. Bulkeley H. (2020). Nature-based solutions for urban biodiversity governance. Environ. Sci. Policy 110, 77–87. 10.1016/j.envsci.2020.04.00235038113

[B154] Zeiher H. (2003). “Shaping daily life in urban environments,” in Children in the City: Home, Neighborhood and Community, editors P. Christensen and O'Brien (London: Routledge Falmer), 66–68.

[B155] Zerlina D. Sulaiman C. C. (2020). Towards the innovative planning for child-friendly neighbourhood in Jakarta. IOP Conf. Ser. Earth Environ. Sci. 592, 012023. 10.1088/1755-1315/592/1/012023

